# Development and assessment of a multiepitope synthetic antigen for the diagnosis of Dengue virus infection

**DOI:** 10.1016/j.bjid.2024.103746

**Published:** 2024-05-01

**Authors:** Isis Botelho Nunes da Silva, Juliano de Moraes Rodrigues, Ramon Cid Gismonti Batista, Vivian dos Santos Gomes, Clarissa de Souza Chacon, Marcius da Silva Almeida, Talita Stelling de Araujo, Bianca Ortiz da Silva, Terezinha Marta Pereira Pinto Castiñeiras, Orlando da Costa Ferreira Junior, Fabiana Avila Carneiro, Monica Montero-Lomeli

**Affiliations:** a Universidade Federal do Rio de Janeiro, Instituto de Bioquímica Médica Leopoldo de Meis, Rio de Janeiro, RJ, Brazil; b Universidade Federal do Rio de Janeiro, Centro Nacional de Biologia Estrutural e Bioimagem, Plataforma Avançada de Biomoléculas, Rio de Janeiro, RJ, Brazil; c Universidade Federal do Rio de Janeiro, Núcleo de Enfrentamento e Estudos de Doenças Infecciosas Emergentes e Reemergentes (NEEDIER), Rio de Janeiro, RJ, Brazil; d Universidade Federal do Rio de Janeiro, Faculdade de Medicina, Departamento de Doenças Infecciosas e Parasitárias, Rio de Janeiro, RJ, Brazil; e Universidade Federal do Rio de Janeiro, Instituto de Biologia, Laboratório de Virologia Molecular, Rio de Janeiro, RJ, Brazil; f Universidade Federal do Rio de Janeiro, Núcleo de Pesquisa (Numpex-Bio), Campus Duque de Caxias Professor Geraldo Cidade, Duque de Caxias, RJ, Brazil

**Keywords:** Dengue virus, Synthetic multiepitope antigens, Diagnosis, Cross-reactivity, Flaviviruses

## Abstract

Immunodiagnostic tests for detecting dengue virus infections encounter challenges related to cross-reactivity with other related flaviviruses. Our research focuses on the development of a synthetic multiepitope antigen tailored for dengue immunodiagnostics. Selected dengue epitopes involved structural linearity and dissimilarity from the proteomes of Zika and Yellow fever viruses which served for computationally modeling the three-dimensional protein structure, resulting in the design of two proteins: rDME-C and rDME-BR. Both proteins consist of seven epitopes, separated by the GPGPG linker, and a carboxy-terminal 6 × -histidine tag. The molecular weights of the final proteins rDME-C and rDME-BR are 16.83 kDa and 16.80 kDa, respectively, both with an isoelectric point of 6.35. The distinguishing factor between the two proteins lies in the origin of their epitope sequences, where rDME-C is based on the reference dengue proteome, while rDME-BR utilizes sequences from prevalent Dengue genotypes in Brazil from 2008 to 2019. PyMol analysis revealed exposure of epitopes in the secondary structure. Successful expression of the antigens was achieved in soluble form and fluorescence experiments indicated a disordered structure. In subsequent testing, rDME-BR and rDME-C antigens were assessed using an indirect Elisa protocol against Dengue infected serum, previously examined with a commercial diagnostic test. Optimal concentrations for antigens were determined at 10 µg/mL for rDME-BR and 30 µg/mL for rDME-C, with serum dilutions ranging from 1:50 to 1:100. Both antigens effectively detected IgM and IgG antibodies in Dengue fever patients, with rDME-BR exhibiting higher sensitivity. Our in-house test showed a sensitivity of 77.3 % and 82.6 % and a specificity of 89.4 % and 71.4 % for rDME-C and rDEM-BR antigens. No cross-reactivity was observed with serum from Zika-infected mice but with COVID-19 serum samples. Our findings underscore the utility of synthetic biology in crafting Dengue-specific multiepitope proteins and hold promise for precise clinical diagnosis and monitoring responses to emerging Dengue vaccines.

## Introduction

Dengue Virus (DENV) is an arthropod-borne virus in the Flavivirus genus of the Flaviviridae family and is primarily transmitted to humans by the hematophagous arthropod *Aedes aegypti*.^1^ According to the World Health Organization, the incidence of dengue has increased dramatically around the world in recent decades, from 505.430 cases in 2000 to 5.2 million cases in 2019.^2^ It is particularly prevalent in the Southeast Asia, the Americas, the Western Pacific, Africa, and the Eastern Mediterranean regions^3^ and travelers around the world can spread this virus to new areas.^4^ Dengue symptoms can range from mild fever (dengue fever) to severe dengue shock syndrome, which can be fatal. The first concern about dengue fever is that only some cases develop harsh symptoms and usually cases are not diagnosed by molecular or sorological diagnosis. This poses an epidemiological problem as cases are underestimated. Another concern is concurrent dengue and zika infections which can cause similar symptoms and can be misdiagnosed clinically. Immunodiagnosis may also pose a cross-reaction between both viruses as they share a high genetic similarity that can lead to misdiagnosis.^5^^,^^6^ Dengue virus shares a 55.6 % amino acid sequence identity with the Zika Virus (ZIKV)^7^ and an even higher identity with Yellow Fever Virus (YFV) and West Nile Virus (WNV).^8^^,^^9^ These viruses belong to the genus Flavivirus which contain a single-stranded, positive-sense genome encoding three structural proteins: Capsid (C), precursor Membrane protein (prM), and Envelope (E) protein, as well as seven Non-Structural (NS) proteins: NS1, NS2a, NS2b, NS3, NS4a, NS4b, and NS5.^10^ The structural proteins are responsible for virus particle formation, with the E protein located on the surface, the M protein providing support, and the C protein associated with genome encapsulation. Additionally, the NS proteins play crucial roles in various stages of both the Dengue and Zika life cycle, including viral replication, capping, virion formation, and viral release.^1^ The four Dengue serotypes (DEN1–4) share an amino acid sequence identity ranging from 60 % to 75 % and can cause the disease.^11^ During evolution, even within the same serotype, viruses can differ by approximately 3 % and 5 % in their amino acid composition and nucleotide composition, respectively, which can alter the sensitivity of immunodiagnostic tests.

Commercial diagnostic tests rely on Enzyme-Linked Immunosorbent Assays (ELISAs) due to their cost-effectiveness and straightforward diagnostic process compared to molecular-based assays or viral isolation. Proteins that induce immune responses in individuals with dengue fever include the Envelope (E) protein, the Premembrane/Membrane (prM/M) protein, and the Non-Structural protein-1 (NS1). NS1 is secreted by infected cells and can be detected in the serum during acute dengue infection.^12^ For this reason, NS1 and E proteins are the primary choice for diagnosing dengue fever in sera patients. Commercial tests, such as the rapid Panbio® Dengue Duo (NS1, IgM and IgG), IgM Capture ELISA (Abbott), or the ELISA plate assay IgG or IgM (Euroimmun) employ IgM/IgG diagnosis, allowing differentiation between primary and secondary infections. The sensitivity of the first in acute phase patients was observed to be 46.8 % with an excellent specificity (94.4 %) in Brazilian patients.^13^ However, there is conflicting evidence regarding the cross-reactivity of these tests with other flavivirus compromising their specific reliability.^14^ Therefore, there is a need to develop cost-effective, safe, and simple diagnostic tests that combine sensitivity and specificity. The initial clinical evaluation is critical, as symptoms can mislead medical doctors, leading to incorrect treatment. To address this issue, we have engineered a multi-epitope antigen using synthetic biology tools and developed a serological test that detects the presence of dengue specific IgM and IgG without cross-reaction to Zika-infected sera and that can be readily re-tailored to new emerging DENV.

## Material and methods

### DENV, ZIKV, and YFV sequence retrieval

We compiled the proteome sequence data from DENV, ZIKV, YFV, Japanese Encephalitis Virus (JEV), and Tick-Borne Encephalitis Viruses (TBEV), isolated from Brazilian patients between 2008 and 2019. The accession codes for these strains are the following. DENV strains: AGK36289.1; AGK36290.1; AGK36293.1; AGK36296.1; AGK36295.1; AGK36299.1; QGW05368.1; QGW05372.1; AGK36292.1; AGN94882.1; QGJ02362.1; ACY70783.1; QGW05381.1. ZIKV virus: A077RB6; AND01116; AMQ48982; AMQ48981; AYV74919; AYV74931. YFV: AWB14994; QDA77104; QBK94341; QBK94342; ATN45408; AVQ67777; ANH10698; AWB14997; AWB15001; AWB15003.JEV and TBEV NP_059434.1 and NP_ 043,135.1, respectively.

### Selection of epitopes

We collected epitopes reported in the literature to design a synthetic dengue multiepitope protein. Rao et al.^15^^,^^16^ described 15 linear IgG specific dengue virus epitopes that were identified through Pepscan analysis, phage display, and computer predictions. The reported epitopes originate from the following proteins: E-DENV2 (8 epitopes), NS1-DENV1 (6 epitopes), NS1-DENV2 (1 epitope), NS2-DENV4 (4 epitopes), and NS3-DENV4 (1 epitope) (Table S1). In a subsequent iteration of reported epitopes, we also selected an epitope derived from protein E from DENV1-4 with the sequence YENLKYTVIITVHTGDQH, which was reported to differentiate between Zika and dengue-infected patients in peptide-based ELISA experiments^17^ (Table S1). After the initial epitope selection, to assemble a multiepitope protein that is optimized to the Brazilian population, epitopes were compared to the proteome of Brazilian strains by multiple sequence alignment tool on the Clustal online server (https://www.ebi.ac.uk/Tools/msa/clustalo/). Epitopes that displayed differential residues between Brazilian strains and those previously reported were chosen. These epitopes were also compared to the corresponding sequence in the proteome of ZIKV and YFV (Table 1).

**Table 1:** Multiple sequence alignment of selected epitopes from DENV Brazilian (rDME-BR) and Asian strains (rDME-C) with the most conserved sequence found in Zika Virus (ZIKV) or Yellow Fever Virus (YFV).

Epitope	Protein of origin	Multiple sequence alignment	
Ep1	ENV	DENBR	ETLVTFKNPHAKKQDVVVLGS 21
		DENC	ETLVTFKNPHAKKQDVVVLGS 21
		ZIKV	EALVEFKDAHAKRQTVVVLGS 21
		YFV	HHLVEFEPPHAATIKVLALGN 21
			.* * *: * * * : . * * .
Ep17	ENV	DENBR	PENLEYTIVITPHSGEEH 18
		DENC	YENLKYTVIITVHTGDQH 18
			* * * : * * : :* * * : * : : *
Ep3	ENV	DENBR	PFGDSYIIIGVE 12
		DENC	PFGDSYIIIGVE 12
		ZIKV	PFGDSYIVIGVG 12
		YFV	PFGDSYIIVGTG 12
			* * * * * * *: : * .
Ep9	NS1	ZIKV	EEEKEWKT 8
		YFV2	EEQEQWKT 8
		YFV1	GLQYGWKT 8
		DENBR	EHKYSWKT 8
		DENC	EHKYSWKS 8
			: * * :
Ep10	NS1	DENBR	DSGCIVSWKNKELKC 15
		DENC	DSGCVVSWKNKELKC 15
		ZIKV	DVGCSVDFSKKETRC 15
		YFV	DQGCAINFGKRELKC 15
			* * * : . : : : * : *
Ep11	NS1	YFV	SYYPEDPVKLASIVKAS 17
		ZIKV	KYHPDSPRRLAAAVKQA 17
		DENBR	KFQPESPAKLASAILNA 17
		DENC	KFQPESPARLASAILNA 17
			. : * : . * : * * : : :
Ep13	NS1	DENBR	FLIDGPETSECPNERRA 17
		DENC	FLIDGPDTSECPNERRA 17
		ZIKV	FVVDGDTLKECPLKHRA 17
		YFV	FIIDGKSRKECPFSNRV 17
			* : :* * . * * * . . * .
Ep14	NS1	YFV	WYPMEIRPKKTHDSHLV 17
		DENBR	WYGMEIRPLSEKEENMV 17
		DENC	WYGMEIRPLSEKEENMV 17
		ZIKV	WYGMEIRPRKEPESNLV 17
			* * * * * * * . : . : : *
Ep15	NS3	YFV1	-ILMMLVSVAGRVDGLELRKL 20
		YF3	-TLEADVILPIGTRSVETDKG 20
		YF2	-CFEGPEEHEILNDSGETVKC 20
		ZIKV	-IMEDSVPAEVWTRHGEKRVL 20
		DENBR	ILEEN-VEVEIWTKEGERKKL 20
		DENC	-ILEENMEVEIWTREGEKKKL 20
			*

### Conservancy and structural alignment of epitopes

To evaluate the potential cross-reactivity with other flaviviruses, the homology of the chosen epitopes was analyzed using the Epitope Conservancy Analysis tool, available in the Immune Epitope Database ‒ IEDB (http://tools.iedb.org/conservancy/).^18^ The identified epitopes were compared with the previously selected proteins of YFV and ZIKV strains (Table 2).

**Table 2:** Linear Epitope sequence conservancy analysis.

Epitope	Fraction of sequences identity ≥60 % (%)	Minimum identity (%)	Maximum identity (%)
Ep1	78.05	42.86	71.43
Ep3	100	75.00	83.33
Ep9	None	37.50	50.00
Ep10	None	46.67	46.67
Ep11	None	41.18	47.06
Ep13	None	47.06	52.94
Ep14	78.05	52.94	76.47
Ep15	None	35.00	45.00
Ep17	None	38.89	44.44

Structural alignments between the secondary structure of DENV epitopes and the corresponding sequence in the ZIKV proteome were performed in the PyMOL 2.4.0 software.^19^ The proteins used as reference were DENV (PDB: 1OKE) and ZIKV (PDB: 5JHM). The selected epitopes were mapped within these sequences and used for the alignment. The Root Mean Square Deviation (RMSD) of atomic positions was used to verify structural conservation between epitopes. The epitopes that both failed the following criteria were discarded: RMSD value lower than 0.3 Å and sequence identity higher than 60.0 % between ZIKV and YFV in the conservancy analysis.

### Engineering of multiepitope synthetic proteins

The epitopes were grouped according to the protein of origin, in the following order: Envelope, NS1, and NS3. The selected epitopes were joined together with the linker GPGPG. At the amino-terminal four additional residues (MGGS) were added for cloning purposes. Finally, an RSHHHHHH polyhistidine tail was added at the carboxyterminal of the protein to enable purification by Immobilized Metal ion Affinity Chromatography (IMAC).^23^

### Modeling of multiepitope synthetic proteins by bioinformatic methods

The secondary and tertiary structure of the DENV multi-epitope sequences was predicted with the PSIPRED server (http://bioinf.cs.ucl.ac.uk/index.php?id=779)^20^ and by using the default parameters from Github at AlphaFold1 tool^21^ (https://colab.research.google.com/github/deepmind/alphafold/blob/main/notebooks/AlphaFold.ipynb). The generated structures were validated by constructing a Ramachandran plot using the online tool Ramachandran Plot Server (https://molprobity.biochem.duke.edu/accesedon 06/14/2023)^22^ to verify if the residues of the protein model are located in allowed regions of torsion angle values.^26^ The hydrophobicity map was analyzed using the Kyte-Doolittle scale to identify accessible epitope regions recognized by antibodies utilizing the online software PROTSCALE (https://web.expasy.org/protscale/).^27^ We used a 9 residues frame, which is suggested by Kyte-Doolittle to evaluate the surface regions of globular proteins (KYTE; DOOLITTLE, 1982). The molecular mass and Isoelectric point (pI) of the sequences used were predicted using ExPASy tools (https://web.expasy.org/protparam).^28^

### Expression and purification of multiepitope proteins

The *E.coli* codon-optimized nucleotide sequences corresponding to the designed rDME-C and rDME-BR proteins were codon-optimize using the Benchling codon optimization tool (https://www.benchling.com/) and cloned into the pET-28a(+) vector with enzymes NcoI and *Bam*HI. The plasmids pET28a+-DMEC and pET28a+DMEBR were synthesized by Genscript Biotech Corp. *E. coli* BL21 (DE3) competent cells were transformed by the heat shock method and selected on LB plates containing 30 μg/mL kanamycin.^23^ After the selection of transformed cells, the expression of multiepitope proteins was initiated with a 20 mL LB starter culture grown overnight at 37 °C, and used to inoculate 250 mL of LB containing 30 μg/mL kanamycin. Expression was induced by adding 1.0 mM IPTG at OD600 = 0.5‒0.8 for 4 h at 37 °C Cells were harvested and resuspended in lysis buffer (50 mM Na_2_HPO_4_, pH 8.0, 300 mM NaCl, 20 mM DTT, 100 mM Imidazole, 1 U DNase, and 1 × Cell lytic™B (SigmaAldrich). The suspension was transferred to a sonication tube and sonicated on ice using 10-second pulses at 10 Watts until the suspension became clear (approximately 5 min). The suspension was cleared by centrifugation at 3000 g and supernatants were filtered through 0.45 µM nitrocellulose membrane and then loaded in a 5 mL HisTrap™ High-Performance column (Cytiva) equilibrated with buffer A (50 mM Na_2_HPO_4_, 300 mM NaCl, 20 mM Dithiothreitol (DTT), and 100 mM imidazole, pH 8.0). Proteins were eluted using the same buffer supplemented to 200 mM and then to 400 mM imidazole. Protein concentration of eluted fractions was confirmed by Bradford assay (Scienco ‒ 210,045) and purity and identity of the eluted fractions was confirmed by Coomassie-blue stained 15 % SDS-PAGE and Western blot analyses. Anti-his tag primary antibody and HRP-labeled secondary antibody were used for Western blots. Western Blots were developed with a chemiluminescent ECL reagent (Thermo-Fisher).

### Fluorescence and light scattering measurement

Intrinsic fluorescence and light scattering measurements were recorded using a Hitachi F-4500 Fluorescence Spectrophotometer. Intrinsic fluorescence was measured by exciting samples at 280 nm and collecting emission between 300 and 420 nm. Light scattering was measured at 90° in the spectrofluorometer by selecting the same wavelength for both excitation and emission (280 nm).

### Immunoindirect ELISA

Assays were performed as follows: 96-well ELISA plates were coated with 60 µL of synthetic proteins (10‒40 µg/mL) diluted in coating buffer (15 mM Na_2_CO_3_ and 35 NaHCO_3_) overnight at 4 °C. Afterward, plates were blocked with 100 μL of blocking buffer (PBS pH 7.4; 0.05 % Tween 20, and 4 % bovine serum albumin). This was followed by three washes with PBS-T followed by the addition of 80 μL of primary antibody (human or mouse serum) and incubation for 2 h at room temperature. After three washes, the assay was revealed with 50 μL of a 1:2000 solution of peroxidase-labeled secondary antibody (anti-human IgG, anti-human IgM, or anti-mouse IgG (Sigma-Aldrich A0170, A6907, and A0168 respectively) for 1 hour at room temperature. The assay was revealed by incubation with 50 µL of Tetramethylbenzidine (TMB) ELISA Substrate (Thermo Scientific, VB 296,364) for 15 min at room temperature. Subsequently, 50 µL of 2 M HCl was added. The plate was read using an ELISA Microplate reader (Spectramax M5 Molecular Devices) at 450 nm. Human samples were measured in triplicate in two to three independent experiments. Samples were categorized as positive or negative when two or more independent experiments were above or below the cutoff value, respectively. Results were recorded quantitively by absorbance at 450 nm. As a golden standard, the commercial Dengue virus IgG-ELISA NS1 and IgM Dengue Type 1‒4 tests (Euroimmun, Lubeck, Germany catalog numbers EI 266b-9601 and EI 266a-9601–1, respectively) were employed. The assays were conducted according to the guidelines provided by the manufacturer. The results for IgM and IgG were determined by calculating the Optical Density (OD) ratio between the human sample and the calibrator sample. The results for IgM and IgG were determined by calculating the Optical Density (OD) ratio between the human sample and the calibrator sample. Positive samples (ratio ≥ 1.1), undetermined (ratio ≥ 0.8‒< 1.1), and negative samples (ratio < 0.8).

### Clinical samples

Dengue serum samples were collected at the Diagnostic Center of Núcleo de Enfrentamento e Estudos de Doenças Infecciosas Emergentes e Reemergentes Screening and Diagnostic Center (NEEDIER) located at Universidade Federal do Rio de Janeiro following a protocol approved by the National Ethics Committee (CONEP, Brazil; n° 63,237,922.0.0000.5257). Subjects who voluntarily presented from February 2023 to February 2024, 2‒15 days after the onset of symptoms related to dengue virus infection (such as fever, headache, extreme tiredness, vomiting, or diarrhea) were interviewed, provided consent, and completed a questionnaire containing demographic data, onset and type of symptoms, gender, age, and previous dengue infections. Venous blood samples were collected, and serum was separated and stored at −30 °C until used. From 42 patients analyzed, 23 tested positive and 19 tested negative in the Euroimmun Anti-Dengue Virus Type 1‒4 (IgM) kit performed by the reference Molecular Virology Laboratory (LVM) at Biology Institute, UFRJ. Furthermore, 10 positive COVID-19 human samples collected in February 2024 were analyzed. These samples were tested by RT-qPCR, by LVM using primers N1, N2, and RP with Ct values ranging from 14 to 28. The onset of respiratory symptoms was between 1‒4 days. Serum from Zika-infected mice on postnatal day 3 with 10^6^ PFU of ZIKV Pernambuco strain was generously provided by Prof. Julia Clarke (Instituto de Ciências Biomédicas, UFRJ).

### Statistical analysis

Data were analyzed with GraphPad Prism (version 8.1.1; GraphPad Software). Results show the average of the data in most of the experiments except when indicated differently. The Cohen´s Kappa value was calculated as *k* = (p_o_ – p_e_) / (1 – p_e_), where p_o_ represents the relative agreement between the two data sets tested and p_e_ represents the probability that the sets agreed purely by chance.^24^

## Results

### Selection of epitopes for modeling a DENV2 multiepitope protein

To design a synthetic multiepitope protein to diagnose Brazilian Dengue-infected sera (rDME-BR) without cross-reaction with ZIKV and YFV, we employed the Design, Build, Test, and Learn (DBTL) cycle to select B-cell epitopes capable of specifically recognizing dengue antibodies. B-cell epitopes were chosen over T-cell epitopes as the objective was to detect IgM or IgG antibodies in patient sera. Linear B-cell epitopes were preferred over conformational epitopes, as the latter can lose their three-dimensional structure in a multiepitope protein. After collection of known we selected those with the highest identity to dengue Brazilian strains circulating between 2008‒2010, but with the lowest identity to Asian strains. To prevent cross-reactions with the main flaviviruses circulating in Brazil, ZIKV and YFV, we analyzed amino acid sequence conservation and protein secondary structure of the selected epitopes (Table 1). The conservancy analysis revealed that epitopes 1, 3, and 14 exhibited the highest identity and conservation to the sequences of ZIKV and YFV (Table 2). To determine whether these epitopes should be excluded, we further compared their secondary structure to that of ZIKV proteins. The root-mean-square distances (RMSD) values obtained were as follows: Ep1 (1.636 Å), Ep3 (0.220 Å), and Ep14 (0.235 Å). Due to the low RMSD values for epitopes 3 and 14, and their high identity to ZIKV and YFV, we decided to exclude them from the selection of epitopes. Epitope 1, despite being 100 % identical to the ZIKV sequence, was included in our selection due to its previous characterization as a specific epitope for the Dengue virus.^25^ As a last step, we evaluated the conservancy between the selected epitopes with the JEV and TBEV. Among the selected epitopes, Ep13 and Ep15 exhibited a 70.0 % sequence identity with JEV, whereas Ep13 demonstrated a 94.12 % sequence identity with TBEV. However, it is important to emphasize that TBEV has not been highly prevalent among the Brazilian population up until now and, therefore, was not considered in our analysis.

### Modeling of dengue multiepitope proteins

**Fig. 1:**
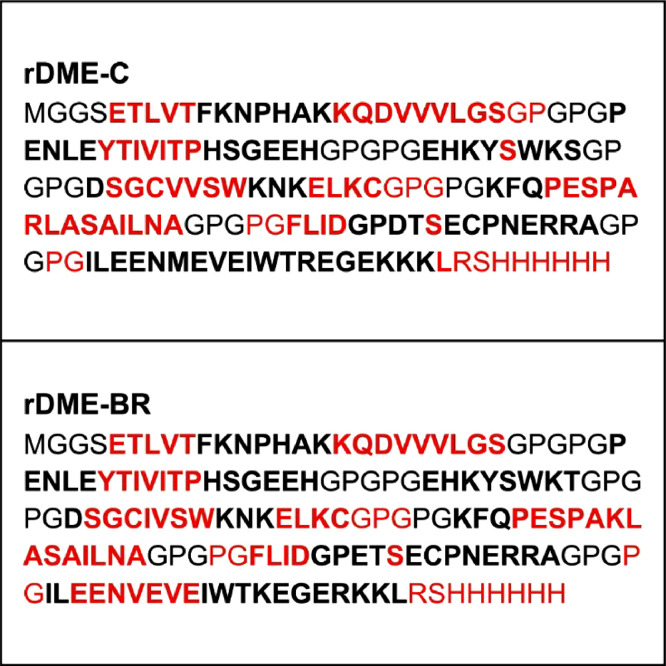
The protein sequence of multiepitope proteins rDME-C and rDME-BR. Epitopes are in bold. Antigenic residues predicted by IEDB (ref) are in red. Epitope order [Ep1] linker [Ep17] linker [Ep9] linker [Ep10] linker [Ep11] linker [Ep13] linker [Ep15]-[6xHisTAG].

Modeling of multiepitope proteins involved the creation of two variants: rDME-C, which maintains the original sequence of the reported epitopes^15^ and rDME-BR, which incorporates conserved mutations specific to Brazilian serotypes (Fig. 1). The arrangement of epitopes within the proteins was meticulously selected to ensure that epitopes originating from the same Dengue protein were neighboring. To join the epitopes, a linker sequence GPGPG was used. This linker sequence has been shown to promote the presentation of epitopes on the protein surface and enhance their interaction with serum antigens.^26^^,^^27^ It has also been reported to be non-immunogenic and confer certain rigidity to the protein, enabling better epitope separation. Additionally, a 6 × Histidine-Tag (His-Tag) was added at the carboxy-terminal of the proteins to facilitate their purification. The final order of the epitopes and the amino acid sequence for each multiepitope protein is presented in Fig. 1. We performed an analysis of the physicochemical properties, as shown in Table 3. Both proteins exhibited an isoelectric point of 6.35, indicating their solubility at pH 7.4, which facilitates their purification under this pH condition.

**Table 3:** Physicochemical properties of multiepitope proteins rDME-C and rDME-BR.

Physicochemical property	rDME-C	rDME-BR
Number residues	158	158
Molecular weight (Da)	16,826.79	16,808.80
Atomic composition	C_739_H_1140_N_216_O_226S5_	C_742_H_1146_N_214_O_226_S_4_
Theoretical PI	6.35	6.35
Extinction coefficient (at 280 nm in H_2_0)	19,480	19,605
Estimated half-life (*E. coli, in vivo*)	> 10 h	> 10 h
Instability index	38.12 (stable)	39.52 (stable)
Grand average of hydropathicity (GRAVY)	−0.840	−0.819
Solubility	Soluble	Soluble

### Secondary and tertiary structure prediction and validation

**Fig. 2:**
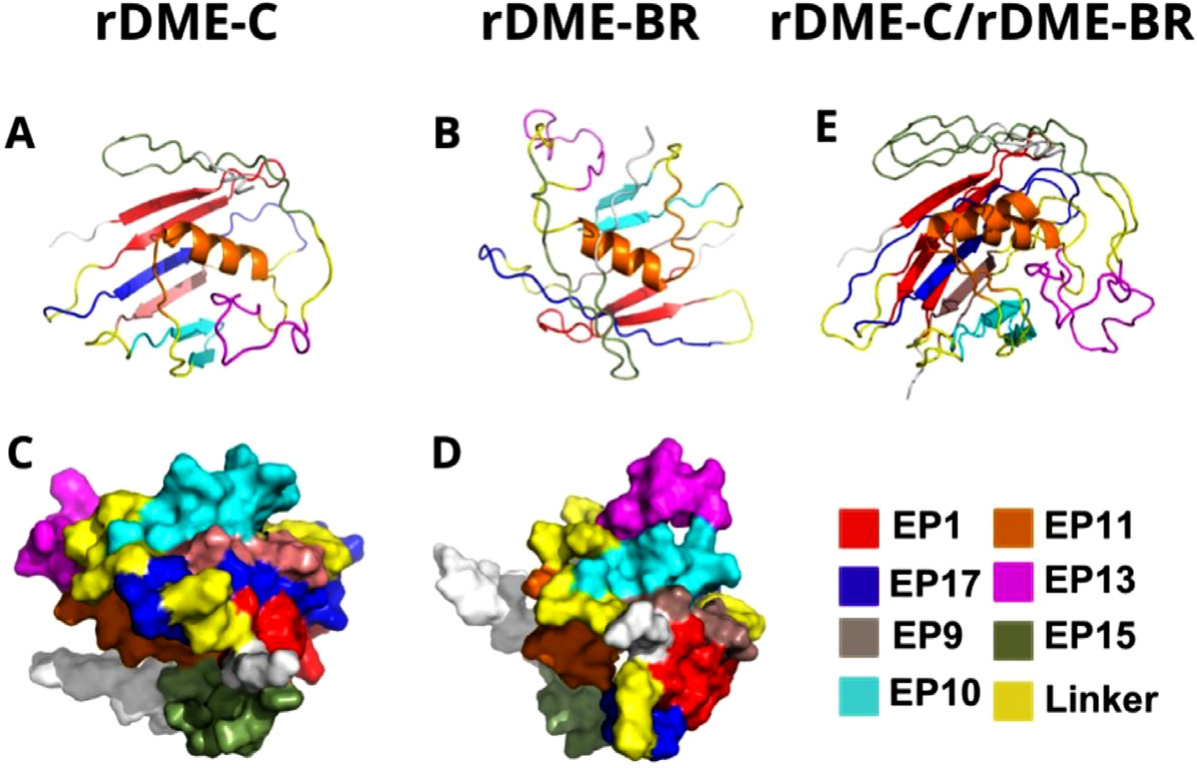
3D structure of rDME-C (A and C) and rDME-BR (B and D). The 3D structure was predicted by AlphaFold1 (ref.) and cartoon (A and B) and surface (C and D) models viewed by PyMol. Epitopes are differentiated by color as shown in the figure. In E, the Alphafold1 cartoon models of rDME-C and rDME-BR proteins were superimposed.

The 2D and 3D structure of the multiepitope proteins was compared between models obtained by Presipred and AlphaFold1. The Root-Mean-Square Deviation (RMSD) difference between the two models was 15.00 and 16.01 for rDME-C and rDME-BR, respectively. This indicates that both software's predictions have high structural differences. To better understand which model would be preferable, we performed Ramachandran plots of the predicted structures by MolProbity software. AlphaFold1 predictions showed a considerably higher percentage of allowed residue positions in contrast to Psipred's predictions (Table 4), so we selected Alphafold1 models (Fig. 2) as the Ramachandran plots predicted a more accurate structure. Importantly, the high exposure of epitopes on the surface of the recombinant proteins and linearity are crucial to enhancing their antigenicity. Analysis of the 2D structure revealed that both proteins are highly unfolded, as 75 % and 83 % of the residues are in the random coil conformation, in rDME-C and rDME-BR, respectively (Table 5). The antigenicity of both proteins was compared using the Kolaskar & Tongaonkar method^28^ with a window size of 7 residues. The antigenic regions predicted are shown in Fig. 1.

**Table 4:** Ramachandran analysis of rDME-BR and rDME-C AlphaFold1 structures.

	rDME-BR		rDME-C	
Feature	Favorable	Allowed	Favorable	Allowed
**AlphaFold1**	80.8 %	96.2 %	81.4 %	96.8 %
**Psipred**	40.4 %	67.3 %	44.8 %	67.9 %

**Table 5:** Secondary structure as in Pymol.

	DME-C	DME-BR
Feature	Amino Acids (%)	Amino Acids (%)
Alpha helix	11 aa (7 %)	11 aa (7 %)
Beta strand	29 aa (18 %)	16 aa (10 %)
Random coil	118 aa (75 %)	131 aa (83 %)

### Expression and purification of rDME-C and rDME-BR

**Fig. 3:**
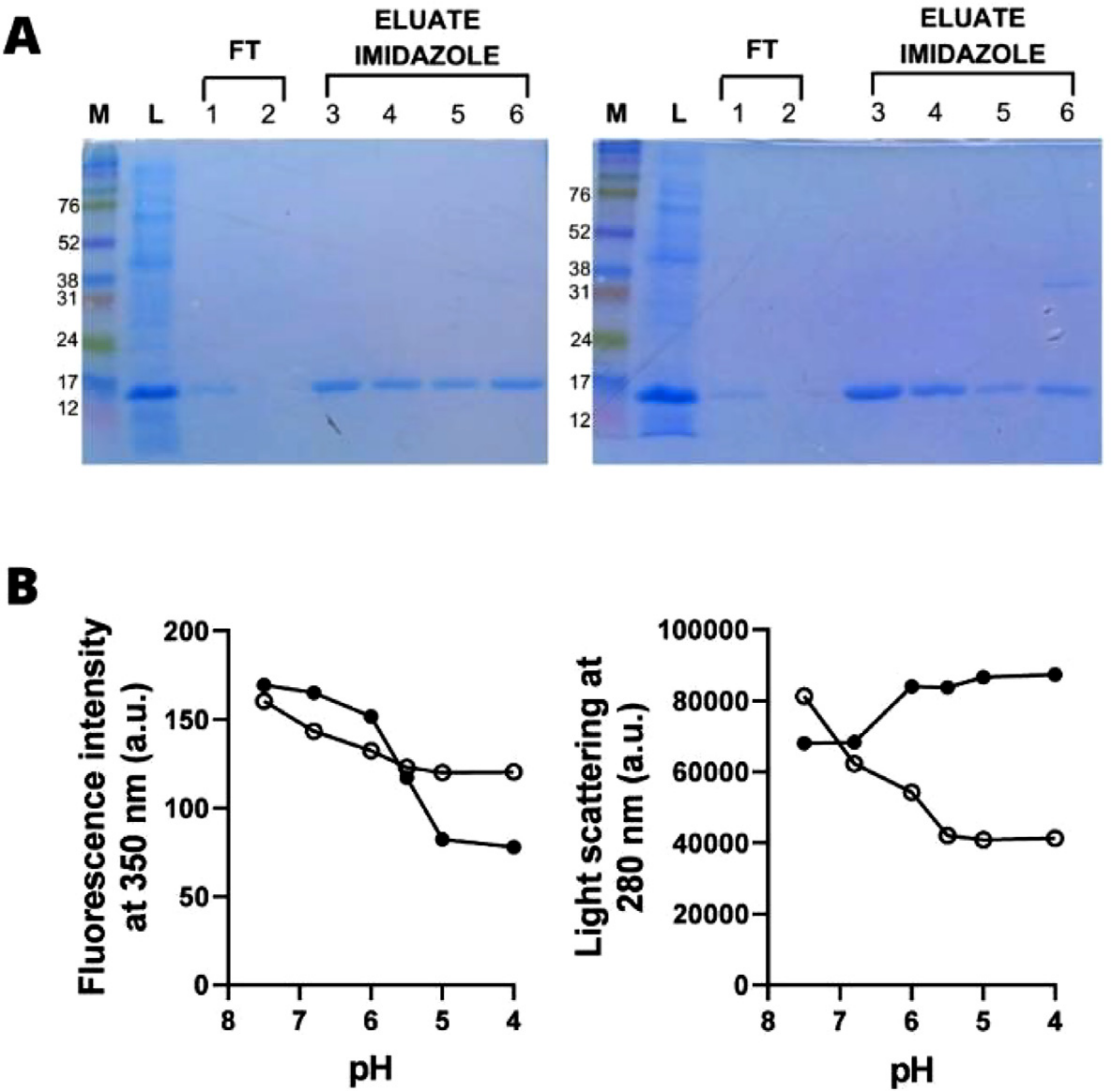
Expression and conformational dynamics of rDME-C and rDME-BR multiepitope proteins. (A) Expression and purification of multiepitope proteins the soluble fraction of rDME-C (left panel) and rDME-BR (right panel) proteins were separated using a 15 % polyacrylamide gel SDS-PAGE The gel lanes were loaded as follows: M (MW marker), L (Lysate),^1^^,^^2^ FT (Flow-through),^3^^,^^4^ Eluate 200 mM imidazole and[5,6] Eluate 400 mM imidazole. (B) Effect of pH on rDME-C (◯) and rDME-BR (•) multiepitope protein. rDME-C or rDME-BR proteins were diluted in phosphate buffer (50 mM, pH 7.5) to a final concentration of 30 µg/mL. (A) Tryptophan fluorescence emission at 350 nm or (B) protein aggregation measured by an increase in light scattering was recorded as pH was gradually acidified by HCl addition. The excitation wavelength was 280 nm in both panels. The data presented are representative of three independent experiments.

The two synthetic multiepitope proteins with apparent MW of 17 kDa, were obtained in soluble form after induction with 1 mM IPTG for 4 hrs. at 37 °C and further purified by affinity His-Tag columns (Fig. 3A and Fig. S1). It should be noted that a protein with a higher molecular weight was also observed. After treatment with 20 mM DTT, these bands disappeared, suggesting aggregation of the multiepitope proteins (data not shown). The molecular dynamics of multiepitope proteins were assessed by measuring the intrinsic fluorescence spectra of rDME-C and rDME-BR (Fig. 3B). Both proteins exhibited maximum emission at 350 nm. This indicates that the Tryptophan (Trp) residues within the proteins are exposed to the polar solvent. As the pH was reduced, the intrinsic fluorescence of both proteins decreased, indicating changes in the environment surrounding the Trp-residues during acidification. However, it is worth noting that the decrease in fluorescence intensity in rDME-C was more gradual compared to rDME-BR. In the case of rDME-BR, the fluorescence intensity graph suggests a significant conformational change occurring at Ph 6.0, as evidenced by the sharp decrease in fluorescence intensity. Based on light scattering measurements, it was observed that acidification induces aggregation in rDME-BR, whereas no aggregation was observed in rDME-C. The conformational changes and aggregation processes seem to occur simultaneously in rDME-BR, as indicated by the changes in both intrinsic fluorescence and light scattering. The most significant changes in intrinsic fluorescence and light scattering occurred between pH 7.0 and 5.0. In contrast, rDME-C did not exhibit any aggregation since the light scattering decreased until reaching pH 6.0, where it stabilized.

### Immunodetection of dengue antibodies by Elisa assays

**Fig. 4:**
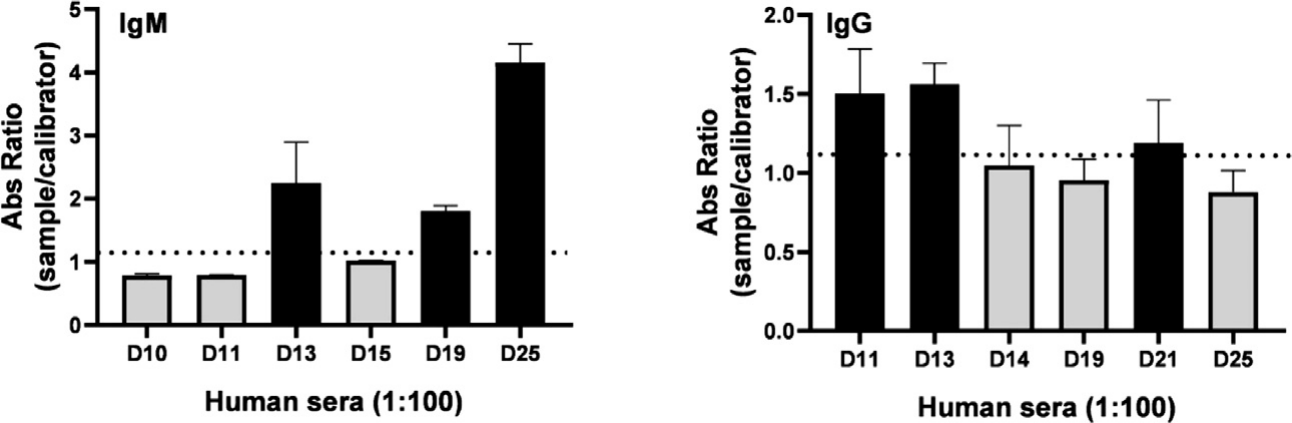
**Determination of positive human IgM/IgG antibodies against dengue virus**. Human serum samples were collected at 6‒7 days of dengue-related symptoms onset, diluted 1:100 and dengue IgM or IgG was determined semi-quantitatively by the commercial test Dengue-Euroimmun. Data presented are mean ± SD as representative of three independent experiments.

**Fig. 5:**
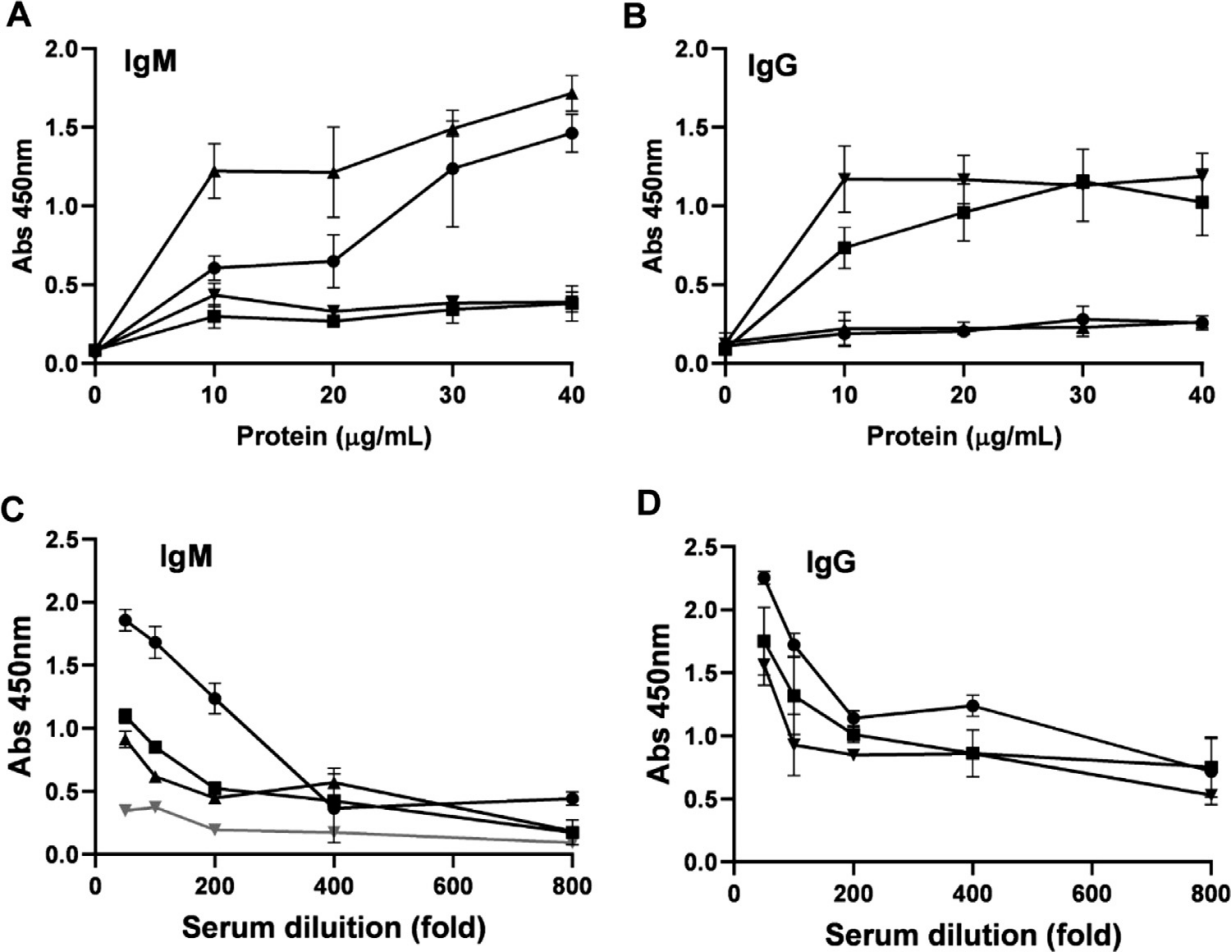
**Determination of human IgM/IgG antibodies against dengue virus**. (A, B) Increasing concentrations of rDME-BR multiepitope protein were used to test dengue IgM (A) or IgG (B) samples at a dilution of 1:100. (C, D) Serum samples were serially diluted from 1:50 to 1:800 and tested against rDME-BR antigen at a concentration of 30 µg/mL. Positive (black lines) or negative (gray lines) IgM or IgG serum samples (Euroimmun test) were used. Data are presented as mean ± SD as representative of three independent experiments.

The collected human sera were tested with the gold-standard anti-IgM and IgG test (EUROIMMUN) (Fig. 4). Three IgM or IgG-positive samples were selected for further tests to assess the reactivity of the rDME-BR and rDME-C antigens. An indirect ELISA assay was used with either anti-human IgM or IgG secondary antibodies for detection. Various concentrations of the antigens (ranging from 10‒40 µg/mL) were tested against positive and negative human sera infected with the DENV (Fig. 5). As expected, our observations revealed that as the antigen concentrations increased, the reactivity was higher with both IgM and IgG secondary antibodies (Fig. 5A‒B). Notably, rDME-BR antigen demonstrated the highest affinity towards both IgG and IgM antibodies. For further experiments, considering that rDME-BR demonstrated higher immunoreactivity, we developed an in-house assay utilizing this antigen at a concentration of 30 µg/mL. This concentration was carefully chosen to avoid crossreactivity with other non-specific antibodies. The assay involved coating plates with rDME-BR antigen. Subsequently, the assay was tested with serial dilutions (ranging from 1:50 to 1:800) of three positive IgM and one negative serum. Also, three positive IgG human sera were used (Fig. 5C‒D). Given the circulation of DENV among the Brazilian population over the past two decades, we did not encounter any IgG-negative sera within our sample set, to be used as a control. The results indicated that the optimal dilution range for the sera was between 1:50 and 1:100, as higher dilutions resulted in lower absorbance levels, as expected.

**Fig. 6:**
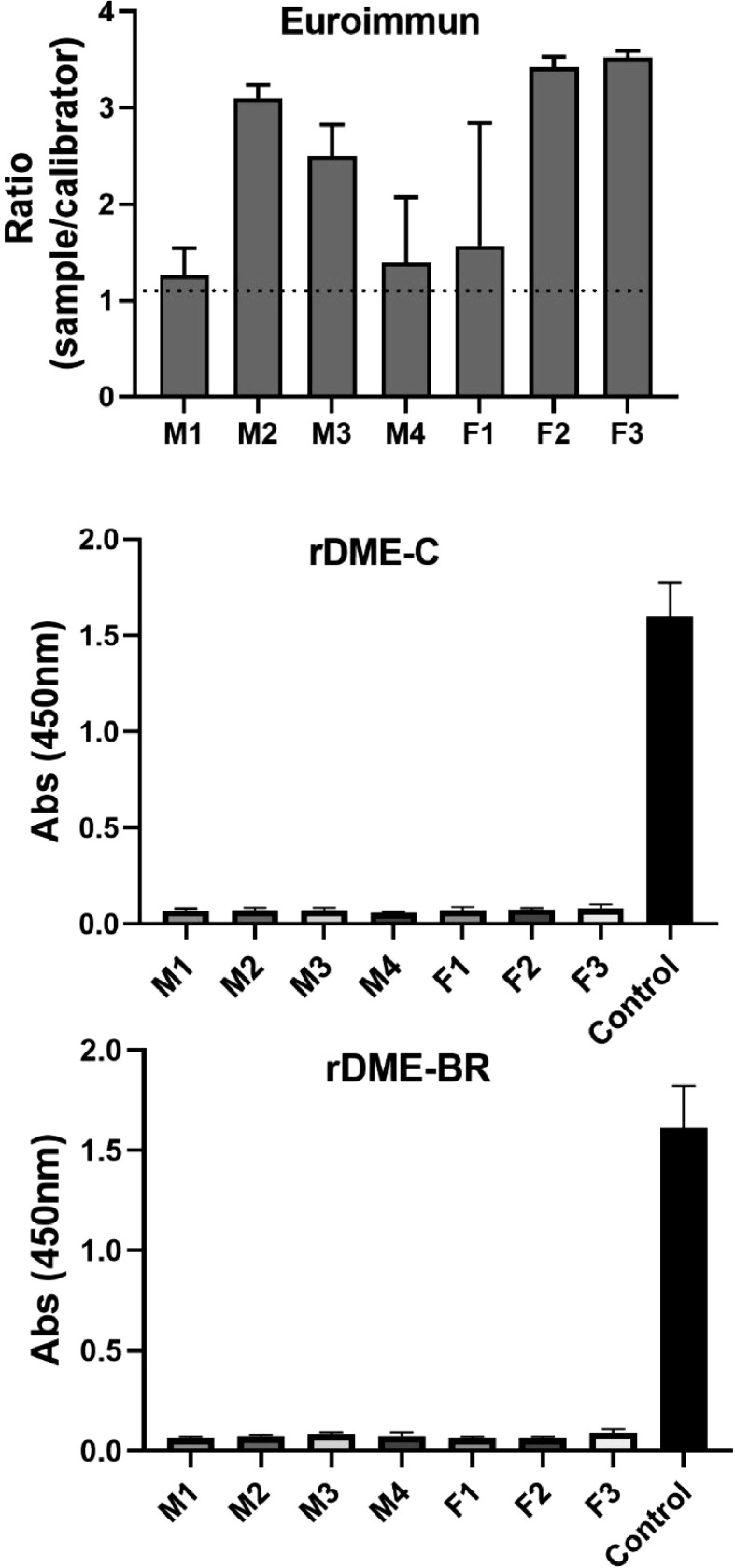
**Determination of cross-reaction between Zika antibodies and rDME-C and rDME-BR.** Serum samples from Zika virus-infected mice (4 males and 3 females) were diluted at a ratio of 1:50 and tested for Dengue using the following: (A) Euroimmun Dengue test; (B, C) Elisa plates coated with rDME-C or rDME-BR multiepitope proteins (30 µg/mL). The ELISA tests were revealed using anti-mouse IgG antibodies labeled with horseradish peroxidase (1:2000). In panels B and C multiepitope proteins were also revealed using anti-His-Tag antibody (1:20,000) (control). The data presented are the mean ± SD of three independent experiments.

Finally, we tested the cross-reactivity of our antigens with Zika antibodies. For this assay, we utilized sera obtained from mice inoculated with the ZIKV (Fig. 6). We compared the commercial test (Euroimmun-Dengue) and our in-house assay. The results demonstrated that the commercial test exhibited reactivity against IgG antibodies from Zika-infected mice. However, our in-house assay did not exhibit any cross-reactivity. This indicates that the use of rDME-BR as Dengue antigen provides specific detection of DENV antibodies in the tested human sera, without interference from cross-reactivity with ZIKV, although the mice antibodies could recognize different epitopes.

### Performance comparison of the in-house indirect Elisa test with a gold standard

Our in-house ELISA tests using either dengue rDME-C or rDME-BR antigens were compared to the commercial anti-Dengue 1‒4 (IgM) Euroimmun kit (Table 6 and Fig. S2). The in-house indirect ELISA test demonstrated a sensitivity of 77.3 % (95 % CI 54.6 % to 92.1 %) and 82.6 % (95 % CI 61.2 % to 95.1 %) for rDME-C and rDME-BR, respectively. The calculated specificity was 89.4 % (95 % CI 66.9 % to 98.7 %) and 71.4 % (95 % CI 47.8 % to 88.7 %) for rDME-C and rDME-BR, respectively. This implies that the rDME-BR antigen had slightly more sensitivity and correctly identified 82.6 % of the positive cases as determined by the gold standard. Comparison of the two antigens rDME-C and rDME-BR show that both could be used for Dengue diagnosis with a positive prevalence value of 100 %, and an accuracy of 77.3 % and 82.6 %.

**Table 6:** Comparison between the in-house indirect Elisa test to a commercial antiDengue Virus Type 1‒4 ELISA (IgM) kit.

Elisa Gold-Standard			In-House indirect Elisa					
Total (n)	Positive (n)	Negative (n)	Antigen	Sensitivity (%) (95 %CI)	Specificity (%) (95 %CI)	PPV (%) (95 %CI)	NPV (%) (95 %C)	Accuracy (%) (95 %CI)
41	22	19	rDME-C	77.27 (54.6‒92.2)	89.4 (66.86‒98.70)	100.00 (82.35‒100)	0	77.27 (61.52‒88.8)
44	23	21	rDME-BR	82.6 (61.2‒ 95.0)	71.42 (47.82‒88.72)	100.00 (86.28‒100)	0	82.61 (88.2‒92.3)

We tested the performance of our in-house Elisa test in these samples. A total of 10 samples from SARS-CoV2 infected sera, previously tested by RT-qPCR showed a cross We tested the performance of our in-house ELISA test in a total of 10 samples from SARS-CoV2 infected sera, confirmed by RT-qPCR. We found a cross-reactivity of 33.3 % using either the IgM-Euroimmun test or the rDME-BR antigen, while it showed a 44.4 % cross-reactivity using the rDME-C antigen.

## Discussion

The importance of specific dengue serological tests that do not cross-react with other arboviruses lies in their ability to provide an accurate and reliable diagnosis of dengue infections. These tests can differentiate DENV from other flavivirus that share similar antigenic properties, such as ZIKVvirus and YFV, which can circulate in each area concomitantly as the same mosquito vectors (*Aedes aegypti* and *albopictus*) are responsible for their transmission. Specific dengue serological tests minimize the risk of misdiagnosis, ensuring appropriate management and treatment for patients. These specific tests can also be used in research, including studies on epidemiology, vaccine efficacy, and immunological responses. Recently, the first Dengue vaccine, CYD-TDV, was licensed, and it will be important to accurately assess vaccine-induced antibody responses to evaluate the effectiveness of dengue control strategies.

Serological Dengue tests commonly employ the NS1 protein as an antigen due to its release into circulation during viral replication and its ability to trigger an immune response. However, other proteins like E and M are also released to circulation and possess immunogenic properties, which could potentially be utilized for diagnosis. These three proteins exhibit a high degree of similarity among flaviviruses, leading to cross-reactivity issues when used as antigens. To address this challenge, multiepitope proteins have gained attention in recent years, particularly in the field of vaccine development. The HIV-1 lipopeptide vaccine serves as an example of such a vaccine and is presently undergoing clinical trials.^29^ Utilizing multi-epitope proteins for diagnosis or vaccine development offers the advantage of preventing cross-reactivity. Epitopes can be selected using bioinformatic tools such as BLAST or ClustalW, which compare genomes and proteomes to identify regions with polymorphisms. Immunogenicity prediction tools like the Immune Epitope Database (IEDB) Analysis Resource aid in identifying T-cell or B-cell epitopes by predicting MHC binding, epitope conservancy, and other immunogenicity aspects.

Computational tools like AlphaFold assist in modeling the structure of multi-epitope proteins. These tools streamline the modeling of synthetic proteins, which can then be experimentally validated.

In this study, we developed two synthetic multiepitope proteins, rDME-C, and rDMEBR, to minimize cross-reactivity with ZIKV and YFV. Initially, we selected 16 epitopes from the literature. After comparing their sequences with other flaviviruses, we narrowed down our selection to 9 epitopes. These epitopes originate from the E, NS1, and NS3 proteins, with 3 from E, 5 from NS1, and 1 from NS3. By incorporating epitopes from multiple Dengue proteins, our antigens offer the potential for enhanced immunogenicity. We also could adapt epitopes to the Brazilian serotypes in circulation within the country.

We compared the structures of both multiepitope proteins. All epitopes are exposed to the protein surface (Fig. 2), which was also evidenced by the immunogenicity of antigens (Fig. 1). In the rDME-BR protein, Epitopes 1 (ENV), 10 (NS1), and 13 (NS1) are more prominently exposed to the surface compared to rDME-C. This structural disparity may explain the different Trp-exposure in rDME-BR protein between pH 7 and pH 5 in fluorescence and light scattering experiments (Fig. 3B). Notably, both proteins share Trp-residues in epitopes 1, 17, 13, and 15, allowing for this comparison.

Multiepitope proteins were soluble after expression which facilitated their use. However, we observed dimers, probably by the formation of disulfide bonds between monomers, as a cysteine residue is present in Ep10 and Ep13 and the dimer was readily converted to their monomeric forms after treatment with DTT. We evaluated if these dimers would prevent antigenicity to DENV-human sera, however, this was not observed (data not shown). We further studied the antigenicity of the multiepitope proteins to Dengue and Zika-infected sera. We compared our in-house indirect ELISA test to the gold standard from Euroimmun and found a specificity of 89.4 % and 71.4 % when rDME-C and rDME-BR antigens were used, respectively. We also found a strong correlation between both assays, with a calculated Cohen´s Kappa value of 0.642 and 0.523 for rDME-C and rDME-BR antigens, respectively, when compared to the commercial test. The discrepancy between both tests could be explained by the different targets of the immunoglobulins present in the sera, as both assays use different antigens. We also show that rDME-BR was more immunogenic than rDME-C to both IgM and IgG antibodies. This may be due to the amino acid adaptation of rDME-BR to the Brazilian serotypes circulating during the last 10 years in the area and to the different 3D structures. For example, Ep15 is more antigenic in rDME-BR than in rDME-C. Also, the presence of more linear epitopes, that own a higher probability of binding to antibodies, are presented in rDME-BR. From the 9 epitopes, 5 presents a secondary structure in rDME-C in comparison to 3 in rDMEBR, which is reflected in the larger number of residues in random coil form in the latter. These factors can account for the highest specificity of dengue antibodies.

It should be noted that both antigens react against IgM and IgG antibodies (Fig. 5). IgM antibodies are present and detectable during the first encounter with the DENV, usually starting from the fifth day after infection. On the other hand, IgG antibodies indicate a past infection, previous vaccination, or secondary contact. As both are recognized, our multiepitope proteins can be used to detect acute infection as well as later stages of infection or used to monitor vaccination rates in a population.

Notably, our multiepitope proteins demonstrated no cross-reactivity with Zika-infected sera but reacted with COVID-19 infected sera. The lack of cross-reactivity with Zika infected sera must be assayed in human infected sera, as mice could recognize different epitopes that are not present in our multi-epitope proteins, but present in the commercial antigens used (protein E from Dengue 1‒4). The high cross-reactivity observed with SARS-CoV2 infected sera has also been observed with other Dengue assays.^30^^,^^31^ At the moment we cannot definitively explain this cross-reactivity, but it is possible that patients could be sequentially infected or co-infected with DENV and SARS-CoV2 virus, as both have been shown to co-circulate in Dengue endemic areas.^32^ In our assays, SARS-CoV2 infected sera that tested positive for Dengue, were positive in both our in-house test and commercial anti-Dengue kit.

Our findings highlight the effectiveness of synthetic biology in designing specific multiepitope antigens for accurate diagnosis of Dengue. Our in-house ELISA assay could be improved now to develop a rapid lateral flow immunoassay strip for detection of DENV antigen, which can be used readily in clinical and home settings. In conclusion, the multiepitope antigens rDME-C and rDMEBR will pave the way for rigorous testing and validation of this innovative antigen. If successful, the impact on public health efforts to combat dengue fever would be substantial.

### Declaration of generative AI and AI-assisted technologies in the writing process

During the preparation of this work the author(s) Mónica Montero-Lomeli used ChatGPT 3.5, Grammarly and Mendeley to improve English language and cite references. After using these services, the author reviewed and edited the content as needed and take full responsibility for the content of the publication.

## Conflicts of interest

The authors declare no conflicts of interest.

## Appendix Supplementary materials

Image, application 1

Image, image 2
