# Supplementary material for: Development and assessment of a multiepitope synthetic antigen for the diagnosis of Dengue virus infection
Source: Braz J Infect Dis. 2024 May 1;28(3):103746. doi: 10.1016/j.bjid.2024.103746 (PMC11096929; doi:10.1016/j.bjid.2024.103746)
Supplement: Supplementary file 1 [file mmc1.doc]

BJID-D-24-00015_Supplementary Material

**Table S1** Epitopes collected in the literature (15–17).

| **Epítope** | **Protein** | **Sequence** | **Serotype** |
| --- | --- | --- | --- |
| Ep1 | E | ETLVTFKNPHAKKQDVVVLGS | DENV2 |
| Ep2 | E | NLLFTG | DENV2 |
| Ep3 | E | PFGDSYIIIGVE | DENV2 |
| Ep4 | E | QLKLNWFKKGSS | DENV2 |
| Ep5 | E | TAWDFGSLGGVFTSIG | DENV2 |
| Ep6 | E | VIITWIG | DENV2 |
| Ep7 | E | STSLSV | DENV2 |
| Ep8 | E | VTLYLGA | DENV2 |
| Ep9 | NS1 | EHKYSWKS | DENV1 |
| Ep10 | NS1 | DSGCVVSWKNKELKC | DENV2 |
| Ep11 | NS1 | KFQPESPARLASAILNA | DENV4 |
| Ep12 | NS1 | LKYSWKTWGKAK | DENV4 |
| Ep13 | NS1 | FLIDGPDTSECPNERRA | DENV4 |
| Ep14 | NS1 | WYGMEIRPLSEKEENMV | DENV4 |
| Ep15 | NS3 | ILEENMEVEIWTREGEKKKL | DENV4 |
| Ep17 | E | YENLKYTVIITVHTGDQH | DENV1-4 |

**Figure S1. Ion exchange chromatography showing typical chromatograms from an AKTA system using His-TRAP columns.** Sample volumes of 30 mL were applied to 5 mL HisTrap™ High-Performance column (Cytiva). His-tagged proteins (rDME-C and rDME-BR) were eluted using a linear gradient from 100‒400 mM imidazol.


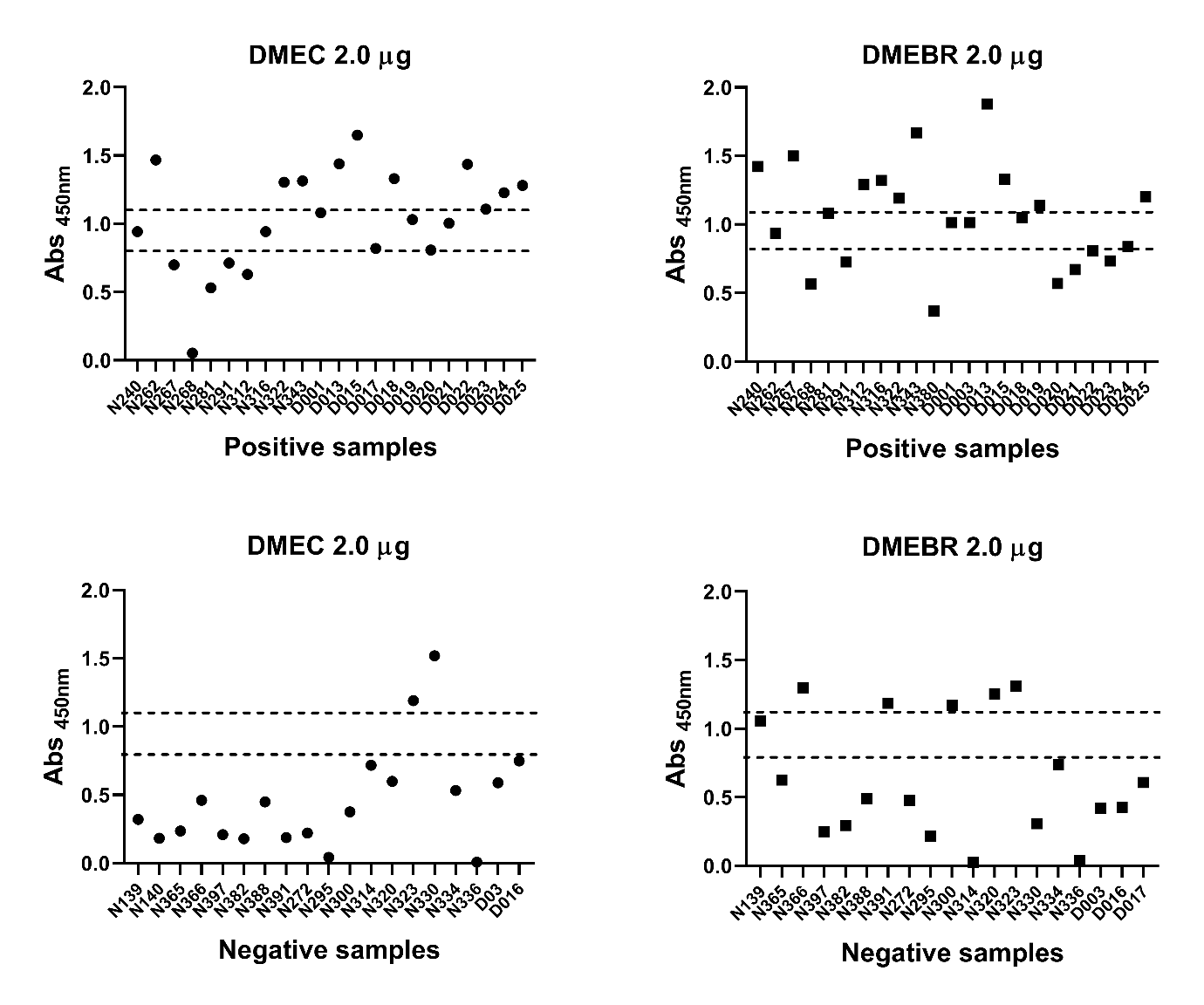


**Figure S2 Absorbance values of in-house rDME-C and rDME-BR indirect Elisa test.** Serum samples from positive and negative Dengue-infected human sera assayed with anti-Dengue 1‒4 kit (Euroimmun) were diluted at a ratio of 1:100 and tested for Dengue using Elisa plates coated with rDME-C or rDME-BR multiepitope proteins (30 µg/mL). The ELISA tests were revealed using anti-mouse IgM antibodies labeled with horseradish peroxidase (1:2,000). Results are absolute absorbance values of the mean of three intra-assay replicates. Cut-off values are represented by a dashed line (Positive samples (Abs_450nm_≥ 1.1), undetermined (Abs_450nm_ ≥ 0.8‒< 1.1), and negative samples (Abs_450nm_ < 0.8).
