# Supplementary figures and images for: Development and assessment of a multiepitope synthetic antigen for the diagnosis of Dengue virus infection
Source: Braz J Infect Dis. 2024 May 1;28(3):103746. doi: 10.1016/j.bjid.2024.103746 (PMC11096929; doi:10.1016/j.bjid.2024.103746)

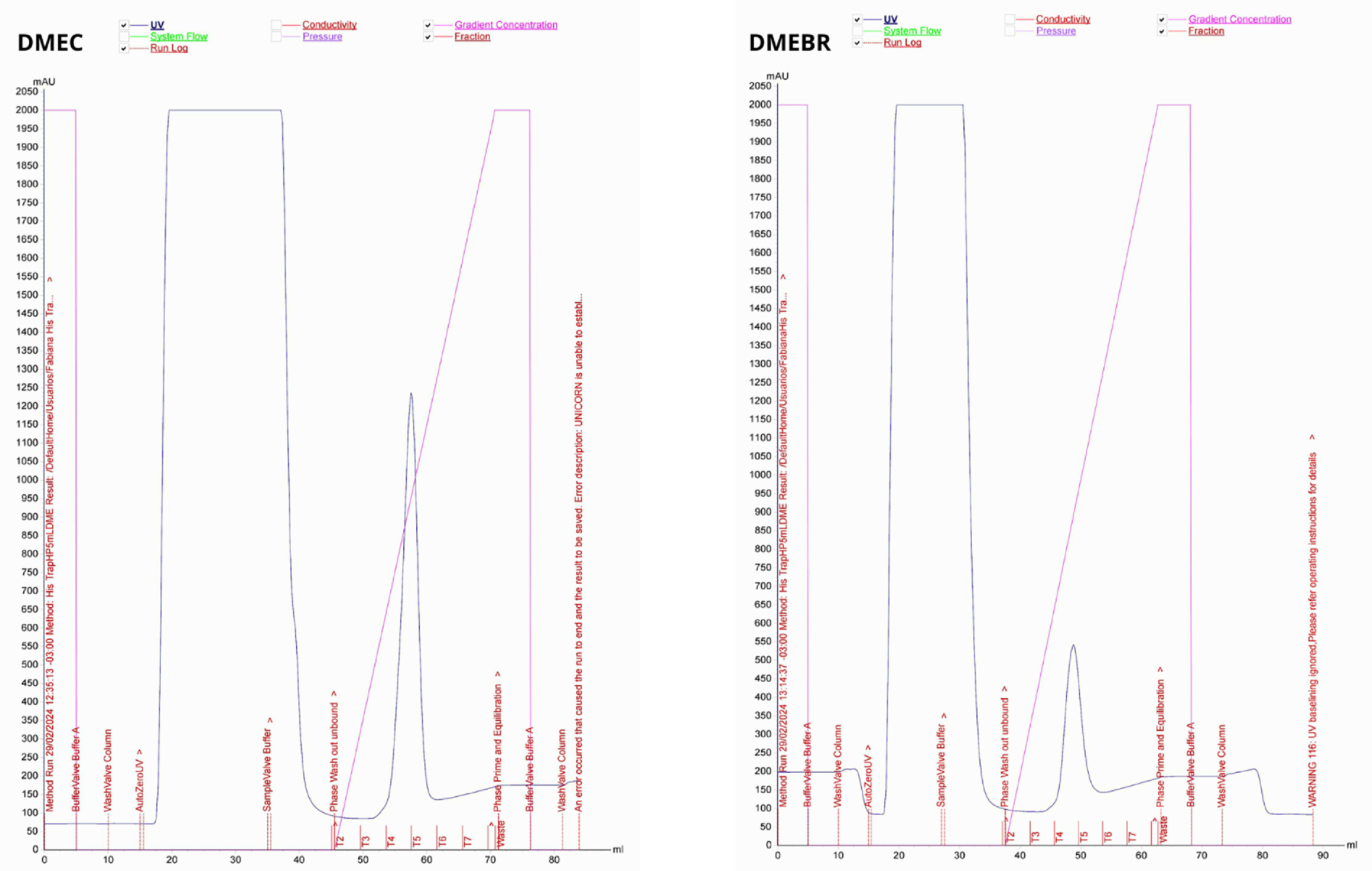

Supplement: Supplementary file 2 [file mmc2.jpg]
